# Tracking Day Shift Nurses’ Work Patterns Using an Indoor Positioning System in an Internal Medicine Ward at a Japanese University Hospital

**DOI:** 10.7759/cureus.97195

**Published:** 2025-11-18

**Authors:** Chifumi Otaki, Shintaro Izumi, Kana Fujimoto, Kyoko Inoue, Miki Nishikawa, Ayumi Teraoka, Izumi Saito

**Affiliations:** 1 Graduate School of Medicine, Kyoto University, Kyoto, JPN; 2 Graduate School of Health Sciences, Technology and Innovation, Kobe University, Kobe, JPN; 3 Department of Nursing, Kobe University Hospital, Kobe, JPN; 4 Department of Public Health, Kansai University of Health Sciences, Osaka, JPN; 5 Department of Maternal Nursing, Tottori College of Nursing, Tottori, JPN; 6 Department of Nursing, University of Tokyo Health Sciences, Tokyo, JPN; 7 Graduate School of Medicine, Kobe University, Kobe, JPN

**Keywords:** dpc/pdps, hospital efficiency, indoor positioning system, japan, nurse staffing, nursing workload, time-motion study

## Abstract

Background: The increasing complexity of medical care and the shortened hospital stays mandated by Japan’s Diagnosis Procedure Combination/Per-Diem Payment System (DPC/PDPS) necessitate the objective and efficient analysis of nursing workload. Given that traditional time-motion studies are resource-intensive, the use of an Indoor Positioning System (IPS) offers an unobtrusive and scalable solution for the continuous quantification of nurse activities.

Objective: This study aims to quantify day-shift nurses’ location-based time-use using an IPS and to evaluate how staffing levels and weekday/weekend status relate to concentrated bedside care and total room presence in a DPC/PDPS ward.

Methods: A prospective time-motion study was conducted in a 51-bed internal medicine ward at a Japanese university hospital for 14 consecutive days between January and February 2025. A total of 153 day shift records were analyzed. Location and duration of stay were measured using smartphones and Bluetooth Low Energy (BLE) beacons. Data were analyzed using Mann-Whitney U tests and Spearman’s ρ.

Results: Nurses spent the largest proportions of time at the nurses’ station (36%) and in patient rooms (30%). The median total patient-room time per nurse was 160.8 minutes (interquartile range (IQR): 111.1-197.2) on weekdays versus 214.5 minutes (147.0-236.2) on weekends (Mann-Whitney U, p = 0.0015). Break-room time was 14.3 minutes (8.3-20.3) on weekdays and 21.8 minutes (12.8-32.0) on weekends (p = 0.0034). At the day level, the number of nurses on duty correlated negatively with the median per-nurse room time (Spearman’s ρ = -0.94, p = 7.2 × 10⁻⁷). Per-patient, bedside time (ward-level) was longer on weekdays than weekends (43.16 minutes (41.33-44.95) vs 28.14 minutes (27.08-28.98); Mann-Whitney U, p = 0.0020).

Conclusions: The IPS effectively quantified nurse location and workload patterns. The finding that total room presence was shorter while bedside time per patient was longer suggests that higher weekday staffing enables more focused and efficient task allocation. IPS technology demonstrates strong practical utility as a scalable, data-driven tool for objectively evaluating nursing efficiency and guiding staffing policy decisions under Japan’s DPC/PDPS system.

## Introduction

In recent decades, the complexity of hospital care has intensified worldwide due to medical specialization, population aging, and increased patient acuity. These trends have expanded nursing responsibilities and increased the urgency of optimizing workload distribution. In Japan, the Diagnosis Procedure Combination/Per-Diem Payment System (DPC/PDPS), introduced to promote shorter inpatient stays, has increased the intensity and documentation burden of nursing work, making objective workload measurement essential [1]. The fixed reimbursement structure of DPC/PDPS incentivizes efficient resource use, though this often necessitates stringent time management and documentation that can potentially divert nurses' time away from direct patient care [2,3].

International evidence consistently indicates that appropriate nurse staffing improves outcomes, including reduced patient mortality and shorter hospital stays [4,5]. However, evidence linking specific staffing models to granular workflow efficiency is limited in Japan, where the need for objective workload evaluation in DPC hospitals has been highlighted [6]. While traditional continuous direct observation (CDO) is the gold standard for workflow quantification [7], it is costly, labor-intensive, and prone to the Hawthorne effect [8,9]. To address these limitations, Indoor Positioning Systems (IPS) and Real-Time Locating Systems (RTLS) offer noninvasive and scalable alternatives [10,11]. IPS technologies, often utilizing Bluetooth Low Energy (BLE) or Wi-Fi signals, enable real-time spatial tracking, providing continuous, nonintrusive data on healthcare worker movement with validated room-level accuracy [12,13].

Prior studies have shown the feasibility of IPS in visualizing nurse movement and work patterns [10,14], and RTLS has been effectively applied in various settings, including emergency departments and operating rooms, to improve process efficiency [11,15]. However, few studies have analyzed the relationship between dynamic staffing levels and care intensity, particularly within Japan’s DPC/PDPS environment [16]. To our knowledge, no previous study has utilized IPS data to examine the association between staffing levels and time-use efficiency under this system.

In this study, IPS-derived metrics are interpreted as indicators of time-use organization rather than direct measures of clinical quality. In particular, concentrated bedside care denotes sustained, per-patient in-room engagement as a proxy for focused task consolidation; it is not, by itself, evidence that longer room time yields superior outcomes.

In a DPC/PDPS hospital context, we used an IPS to quantify day-shift nurses’ location-based time-use and to test how staffing levels and weekday/weekend status are associated with IPS-defined concentrated bedside care (sustained in-room engagement) and total room presence. As secondary aims, we compared weekday versus weekend distributions of these IPS measures and examined the association between daily staffing levels and total room presence.

## Materials and methods

This exploratory prospective observational study was conducted in a 51-bed internal medicine ward at a university hospital in the Kinki region of Japan. Data collection covered 14 consecutive day shifts (08:30-17:15) between January 24 and February 6, 2025, a period with high volumes of documentation, coordination, and direct care under the DPC/PDPS system. Ethical approval was obtained from Kobe University Graduate School of Medicine (B22014-H). All nurses received written study information and provided written informed consent. All rostered day-shift nurses were eligible. Staffing assignments followed the routine rotating roster and were independent of the study; investigators neither influenced scheduling nor selected individuals for observation. Inclusion comprised all day-shift nurses working during the period. Shifts with more than 10% IPS data loss due to battery or connectivity issues were excluded. In total, 153 valid day-shift datasets were analyzed. No new clinical protocols, staffing policy changes, or bed-allocation changes were introduced during the 14-day observation window, as confirmed by nursing administration. To minimize reactivity, the IPS application ran in the background without alerts, prompts, or real-time displays; beacons were fixed and unobtrusive; no performance feedback or incentives were given; investigators did not enter the ward during shifts; and staffing rosters and workflows proceeded as usual. Fourteen consecutive day shifts (including weekends) were observed to attenuate novelty effects. The IPS logs stored only the timestamp, a pseudonymous device identifier, a beacon identifier, and the received signal strength; no patient identifiers were recorded. All datasets were de-identified prior to analysis and stored on secure institutional servers under access control.

**Figure 1:**
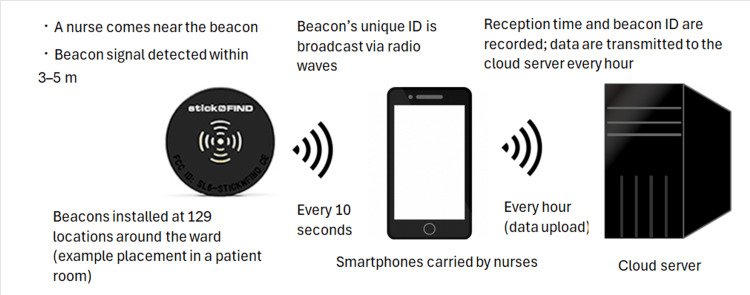
Configuration of the Indoor Positioning System (IPS) for nurse tracking Beacons were installed at 129 ward locations (patient-room entrances, key areas of the nurses’ station, and major hallway intersections). A Bluetooth Low Energy (BLE)-enabled smartphone carried by each nurse detected a beacon within approximately 3-5 meters, logged the reception time and beacon ID, and transmitted records to a cloud server hourly. The system provided continuous estimation of nurse locations and stay durations without interference with medical equipment

Nurse locations were tracked using BLE smartphones (SHARP AQUOS wish SH-M20) carried during routine duty and wireless beacons installed at 129 ward locations, including patient-room entrances, the nurses’ station, and major hallways (the beacon layout is referenced in Figure 1). BLE operated on the 2.4-GHz band, which is safe around medical devices. When a beacon was detected, the device logged timestamp, beacon ID, and received signal strength; proximity logs were uploaded hourly. Beacons broadcast every 10 seconds, and raw logs were aggregated into 10-second epochs. For each epoch, a room-level geofence map (patient rooms, hallway, nurses’ station, treatment room, interview/conference room, break room, bathroom/shower room, other areas) was used to assign a location according to the modal beacon zone. Ties were resolved deterministically in the following order: patient room, nurses’ station, treatment room, hallway, and other zones; if still tied, the zone associated with the higher received signal strength indicator (RSSI) was selected. Epochs without any ward beacon for at least 60 seconds were labeled outside the ward (e.g., pharmacy, radiology). In this study, “bedside stay” is an operational term referring to dwell time detected within the patient-room geofence. Geofences were drawn at room boundaries; doorsill beacons were placed at room entrances so that epochs with patient-room beacons as the modal signal were assigned to the patient-room zone rather than the nurses’ station. Because IPS provides room-level accuracy (approximately 3-5 meters), it cannot resolve microlocations within a room. To reduce transient misclassification at thresholds, we required at least 10 seconds of continuous dwell to register a bedside arrival, and we applied deterministic tie-break rules that favored the patient-room zone over adjacent areas when signals were equivalent.

**Figure 2:**
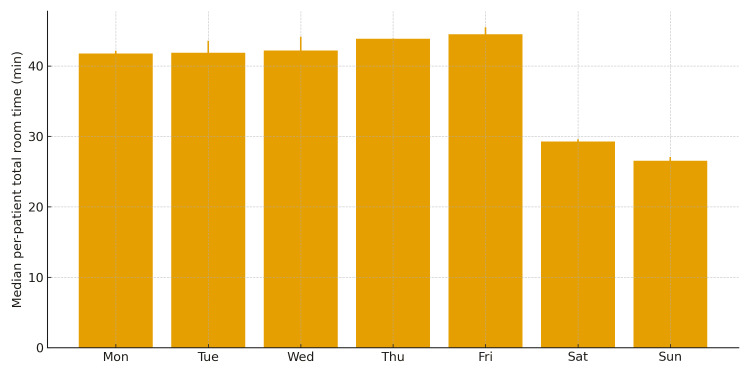
Ward-level per-patient bedside time by weekday vs weekend/holiday (day-level comparison) IQR: interquartile range For each day, per-patient bedside time was computed as Σ (all nurses’ patient-room minutes) divided by the inpatient census. Values shown are medians (IQR) across days. Two-sided Mann-Whitney U (α = 0.05) compares day-level distributions

Consecutive epochs assigned to the same zone were merged into dwell intervals. To exclude transient pass-bys at doorways, bedside arrival in a patient room required at least 10 seconds of continuous dwell. Location-based times were computed as the sum of dwell durations per zone and expressed in minutes. Unless otherwise specified, location-based dwell times are summarized per nurse per day shift (median minutes). In addition, a ward-level per-patient bedside time was derived as the sum of all nurses’ patient-room minutes per day divided by the inpatient census (minutes per patient per day). This per-patient metric is not a per-nurse value and is used in the weekday-weekend comparisons and in Figure 2. Total patient-room time was defined as the total minutes each nurse spent in patient rooms during a shift. The ward-level per-patient bedside time followed the definition above. As a sensitivity analysis, “concentrated bedside care” at the visit level was operationalized as the median dwell per in-room visit for each patient on a given day, summarized at the ward level by the median across patients; results for this sensitivity metric are not tabulated in this manuscript but showed the same qualitative weekday-weekend pattern as the ward-level metric.

In this ward, routine vital signs were obtained in patient rooms using non-networked bedside devices, and there was no capability to acquire new physiologic measurements from the nurses’ station. Activities at the station comprised electronic health record documentation and order entry, coordination and telephone triage, medication preparation and verification, handovers, and logistics. By geofence definition, any activity delivered inside a patient room, including assistance with activities of daily living, toileting, grooming, patient or family communication, and bedside education, was classified as patient-room dwell (direct bedside time), whereas station time reflected indirect care. The analytic scope was limited to day shifts; within-shift temporal stratification and evening or night shifts were not analyzed in this study and are identified as future work.

Shifts with more than 10% missing IPS data were excluded. Device clocks were checked daily against the hospital time server and showed no systematic drift. Isolated single-epoch spikes not contiguous with adjacent assignments, for example, after brief reboots, were flagged and removed. Daily operational metrics, including inpatient census, nursing intensity/acuity scores, and staffing ratios, were obtained from the hospital’s electronic records. Weekday/weekend status was coded at the day level according to the hospital calendar, with weekdays defined as Monday to Friday and weekends/holidays as Saturday, Sunday, and public holidays. All analyses used day-shift records (08:30-17:15).

Medians with interquartile ranges were reported for all summaries. Two-sided Mann-Whitney U tests (α = 0.05) compared weekday versus weekend/holiday day shifts. Spearman’s ρ assessed day-level associations between nurse staffing counts and the median per-nurse total patient-room time as well as the ward-level per-patient bedside time. Analyses were performed in IBM SPSS Statistics for Windows, Version 29 (Released 2022; IBM Corp., Armonk, New York, United States).

## Results

**Figure 3:**
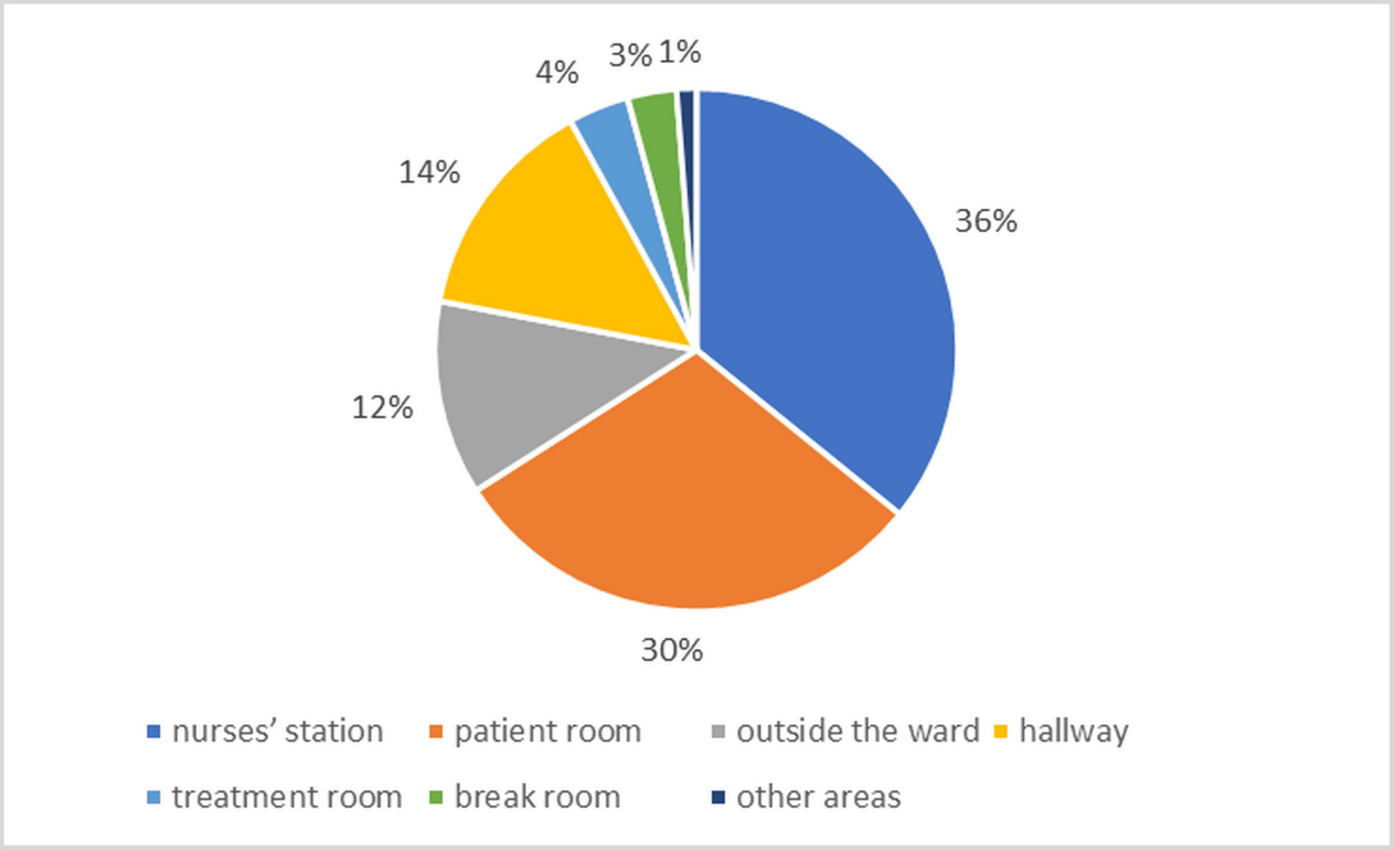
Distribution of nurses’ total working time by location (N = 153 nurse-shift datasets) Nurses spent 36% of total working time at the nurses’ station and 30% in patient rooms; the remaining time was distributed among the hallway (14%), outside the ward (12%), treatment room (4%), break room (3%), and other areas (1%)

A total of 153 valid day-shift nursing datasets were analyzed. The number of inpatients per day ranged from 40 to 51, and the number of day-shift nurses varied across the 14 measurement days (Table 1). Of the 153 day-shift datasets, 128 (83.7%) were weekdays and 25 (16.3%) were weekends/holidays. Nurses spent the largest proportion of time at the nurses’ station (36%) and in patient rooms (30%). Time in other areas included the hallway (14%), outside the ward (12%), treatment room (4%), break room (3%), and other areas (1%). Figure 3 illustrates this distribution, showing a concentration of activity between the nurses’ station (coordination and documentation) and patient rooms (direct care). The 153 nurse-shift records were contributed by nurses scheduled per routine rosters across the 14 consecutive days.

**Table 1:** Patient, nurse, and measurement period data Daily patient census, day-shift nurse staffing, and observation days are summarized. The 14 consecutive day-shift observations yielded 153 valid shifts: 128 (83.7%) weekdays and 25 (16.3%) weekends/holidays. Daily ranges of census and staffing across the observation period are presented

Date	Day of the week	Total number of patients	Number of day shift nurses
24-Jan	Tue	40	10.5
25-Jan	Wed	41	11
26-Jan	Thu	44	10.5
27-Jan	Fri	43	12.5
28-Jan	Sat	42	6
29-Jan	Sun	41	6
30-Jan	Mon	44	11.5
31-Jan	Tue	46	11.5
1-Feb	Wed	49	12.5
2-Feb	Thu	51	14.25
3-Feb	Fri	50	15.25
4-Feb	Sat	46	7
5-Feb	Sun	45	6
6-Feb	Mon	45	14.25

The median total time spent in patient rooms per nurse was 160.8 minutes (IQR: 111.1-197.2) on weekdays and 214.5 minutes (147.0-236.2) on weekends (Mann-Whitney U, p = 0.0015). Time spent in the break room was longer on weekends (median: 21.8 minutes; IQR: 12.8-32.0) than on weekdays (14.3 minutes, 8.3-20.3; Mann-Whitney U, p = 0.0034), suggesting a different organizational flow during low-staff periods. Weekday-weekend comparisons for all locations are summarized in Table 2.

**Table 2:** Weekday vs weekend/holiday comparison of location-based dwell times (day shift) IQR: interquartile range Values are median minutes (IQR) per nurse per shift; p-values from two-sided Mann-Whitney U (α = 0.05). Weekdays n = 128, weekends/holidays n = 25

Location	Weekdays (n = 128), median (IQR)	Weekends/holidays (n = 25), median (IQR)	p-value
Patient room	160.8 (111.1-197.2)	214.5 (147.0-236.2)	0.0015
Break room	14.3 (8.3-20.3)	21.8 (12.8-32.0)	0.0034
Interview/conference room	0.2 (0.0-0.3)	0.2 (0.0-0.2)	0.2999
Treatment room	14.4 (9.0-21.1)	11.8 (9.2-22.2)	0.8667
Bathroom/shower room	0.0 (0.0-0.0)	0.0 (0.0-0.2)	0.7620
Hallway	70.6 (58.8-86.2)	79.2 (65.8-86.2)	0.3459
Nurses’ station	188.4 (159.0-206.5)	167.8 (148.8-197.8)	0.2574
Other areas	0.2 (0.0-0.5)	0.5 (0.2-1.3)	0.0216
Outside the ward	33.3 (14.6-81.8)	15.8 (9.2-21.5)	0.0017

For each day, per-patient bedside time was computed as the sum of all nurses’ patient-room time divided by the inpatient census. The median per-patient bedside time was 43.16 minutes (IQR: 41.33-44.95) on weekdays versus 28.14 minutes (27.08-28.98) on weekends/holidays (Mann-Whitney U, p = 0.0020). Figure 2 shows day-of-week medians with IQR for this ward-level metric. These weekday-weekend differences in ward-level per-patient bedside time are presented in Table 3 and were significant by two-sided Mann-Whitney U (p = 0.0020). As summarized in Table 3, ward-level per-patient bedside time was significantly longer on weekdays than on weekends (43.16 vs 28.14 min/day; p = 0.0020), complementing the finding that per-nurse total room time was shorter on weekdays.

**Table 3:** Ward-level per-patient bedside time by weekday vs weekend/holiday (day-level comparison) For each day, per-patient bedside time was computed as Σ (all nurses’ patient-room minutes per day) divided by the inpatient census (minutes per patient per day). Values are medians (IQR) across days. Two-sided Mann–Whitney U test (α = 0.05) compares day-level distributions. Notes: n refers to the number of observation days (14 consecutive days: weekdays = 10, weekends/holidays = 4)

Metric (ward-level)	Weekdays (n = 10 days), median (IQR)	Weekends/holidays (n = 4 days), median (IQR)	p-value
Per-patient bedside time (min/day)	43.16 (41.33-44.95)	28.14 (27.08-28.98)	0.0020

In addition, ward-level time outside the ward showed a higher median on weekdays than on weekends, consistent with weekday diagnostic testing and procedures. The inpatient census ranged from 40 to 51 across the observation window and showed no clear weekday-weekend shift.

At the day level, the number of nurses on duty correlated negatively with the median per-nurse total room time (Spearman’s ρ = −0.94, p = 7.2 × 10^-7^). By contrast, its association with the ward-level per-patient bedside time was weak and nonsignificant (ρ = 0.48, p = 0.0848).

## Discussion

This study demonstrated that the IPS is a valid and unobtrusive method to measure nurse movement, duration, and workload, reinforcing its utility in healthcare workflow analysis [11,17]. The key pattern was a weekday-weekend divergence: on weekdays, per-nurse total patient-room time was shorter, whereas ward-level per-patient bedside time was longer. This ward-level increase in per-patient bedside time on weekdays (Table 3) complements the shorter per-nurse total patient-room time, consistent with more focused in-room engagement when staffing is higher. Our interpretation emphasizes organizational efficiency; that is, when staffing is higher, indirect tasks are more readily consolidated at the nurses’ station so that in-room periods can be more focused. We do not equate total room time with better care, nor do we consider station or break time futile; both reflect necessary components of safe care delivery and staff recovery. We emphasize that the present analyses do not test clinical outcomes. Measures such as length of stay, in-hospital mortality, or healthcare-associated infection rates were not collected and cannot be inferred from IPS location and dwell alone. Accordingly, we do not assert that greater patient-room time necessarily yields better services; the relationship between time allocation and quality may be nonlinear and context-dependent. Conceptually, our weekday-weekend contrast is compatible with the “missed nursing care” framework under staffing constraints [18]. Future studies should link IPS-derived measures with patient-level outcomes and case-mix variables to evaluate whether specific patterns of direct and indirect work translate into measurable improvements in safety and effectiveness. Without task content and fatigue measurement, we cannot determine whether observed dwell differences arise from case mix, task bundling, or fatigue-related pacing. Pre-/post-comparisons or prior-year baselines were not examined and were outside this study’s scope. Future studies should link IPS-derived measures with electronic health records and evaluate length of stay, mortality, and healthcare-associated infections using matched historical periods or concurrent control wards (e.g., interrupted time-series or difference-in-differences) to determine whether specific patterns of direct and indirect work translate into measurable improvements. Taken together, these results indicate associations between staffing levels and IPS-derived time-use patterns at the ward/day level. Given the observational design, lack of task annotation, and no linkage to patient outcomes, these patterns should be interpreted as organizational signals rather than causal effects.

Our framework distinguishes between indirect work that can be organized at the nurses’ station and direct care that must occur in the patient room. In this setting, core assessment and ADL support were inherently bedside, while documentation, coordination, and medication-related preparation were concentrated at the station. IPS location and dwell quantify this organizational allocation but do not encode task content or effectiveness. We did not measure fatigue or stratify dwell by within-shift timeframes, nor did we analyze evening or night shifts; consequently, we cannot determine whether patient-room time remains equally effective under fatigue or whether temporal patterns differ across parts of the shift. Future studies should incorporate validated fatigue or workload instruments, within-shift temporal analyses, and multishift comparisons to test these possibilities while linking to patient outcomes.

The time spent at the nurses’ station (36%) and in patient rooms (30%) aligns with prior time-motion studies [19,20], confirming that documentation, coordination, and administrative activities remain central components of nursing work, a burden exacerbated by the DPC/PDPS system [2,3]. The dynamic data from IPS revealed frequent alternation between the station and patient rooms, highlighting interruptions and multitasking often overlooked by traditional work sampling [19,20]. Weekend staffing reductions coincided with longer per-nurse room presence but shorter per-patient bedside time, suggesting a trade-off between the breadth (general presence) and depth (focused engagement) of care during low-staff periods, an observation that is conceptually consistent with the missed nursing care framework under staffing constraints [18].

A key strength is the continuous, unobtrusive IPS capture of nurse location and dwell across 14 consecutive routine day shifts with room-level coverage via 129 beacons, yielding high ecological validity. Deterministic processing (10-second epoching and pass-by filter, geofence-based zone assignment with prespecified tie-break rules) and predefined quality-control thresholds enable reproducible aggregation without interrupting care. The design leveraged routine rosters with no protocol or staffing-policy changes during observation, reducing selection and implementation bias. Methodologically, we quantify workflow using two complementary interpretable metrics, per-nurse total patient-room time and ward-level per-patient bedside time, and verify weekday-weekend differences using robust nonparametric statistics, with the ward-level per-patient bedside time summarized in Table 3. Together, these features extend traditional time-motion methods by delivering scalable, real-world signals that are immediately actionable for staffing and task allocation while preserving transparency and replicability.

IPS affords room-level, not within-room, localization and does not encode task content; thus, “bedside stay” is an operational proxy for in-room presence that may include brief indirect work (e.g., admission/discharge explanations, brief documentation, preparation). To mitigate doorway misclassification, we applied deterministic zone-assignment rules, a 10-second pass-by filter, and predefined quality-control thresholds, but residual error remains possible. Nurse room time could not be linked to individual patient acuity or outcomes, and the ward lacked remote physiologic monitoring; therefore, “time away from the patient room” did not include remote vital-sign acquisition, and causal interpretation regarding direct-care intensity is limited. The study was observational in a single internal-medicine ward; analyses were restricted to day shifts without within-shift temporal stratification, leaving potential fatigue- or time-of-day effects unmeasured. The observation window comprised 14 consecutive winter days, which may not reflect seasonal or longer-term operational variation in workflow. In addition, the analyses used nonparametric, bivariate tests without multivariable adjustment; thus, potential confounding by factors such as inpatient census, nurse mix/skill level, or case mix could not be controlled statistically, an issue well-recognized in nurse staffing research [21]. These choices reflect the exploratory design and short series; future studies should extend observation across seasons and employ multivariable modeling on longer time series [21]. Pre-/post- or prior-year comparators were not included, so secular trends and a Hawthorne effect cannot be excluded. Although the IPS was passive and introduced no workflow prompts, any residual reactivity, potentially greater on well-staffed weekdays, was not quantified; future studies should incorporate longer acclimatization periods and/or concurrent control wards to estimate this effect. Staffing followed routine rosters with no protocol or policy changes during the 14-day window, yet residual selection bias (e.g., unequal repetition of individual nurses across days) remains possible. These constraints limit generalizability and preclude causal claims; findings should be treated as hypothesis-generating and confirmed in multiward, multiperiod studies that pair IPS with task annotation, validated fatigue measures, and EHR-linked patient outcomes. Classic multihospital time-motion work similarly highlights the substantial share of documentation/coordination in nurses’ day, providing historical context for our IPS-derived distributions [22].

## Conclusions

The IPS successfully quantified nurse workflow patterns in a Japanese university hospital ward. Higher staffing was associated with shorter total time in patient rooms but longer per-patient in-room dwell, findings consistent with a redistribution of indirect work toward the nurses’ station and more focused in-room engagement during bedside contacts. Overall, our conclusions reflect associations observed in routine practice and do not imply causality; task-level content and outcome linkage were beyond the scope of this study and are needed to determine clinical impact.
